# Neurite Outgrowth Mediated by Translation Elongation Factor eEF1A1: A Target for Antiplatelet Agent Cilostazol

**DOI:** 10.1371/journal.pone.0017431

**Published:** 2011-03-01

**Authors:** Kenji Hashimoto, Tamaki Ishima

**Affiliations:** Division of Clinical Neuroscience, Center for Forensic Mental Health, Chiba University, Chiba, Japan; Tokyo Institute of Psychiatry, Japan

## Abstract

Cilostazol, a type-3 phosphodiesterase (PDE3) inhibitor, has become widely used as an antiplatelet drug worldwide. A recent second Cilostazol Stroke Prevention Study demonstrated that cilostazol is superior to aspirin for prevention of stroke after an ischemic stroke. However, its precise mechanisms of action remain to be determined. Here, we report that cilostazol, but not the PDE3 inhibitors cilostamide and milrinone, significantly potentiated nerve growth factor (NGF)-induced neurite outgrowth in PC12 cells. Furthermore, specific inhibitors for the endoplasmic reticulum protein inositol 1,4,5-triphosphate (IP_3_) receptors and several common signaling pathways (PLC-γ, PI3K, Akt, p38 MAPK, and c-Jun N-terminal kinase (JNK), and the Ras/Raf/ERK/MAPK) significantly blocked the potentiation of NGF-induced neurite outgrowth by cilostazol. Using a proteomics analysis, we identified that levels of eukaryotic translation elongation factor eEF1A1 protein were significantly increased by treatment with cilostazol, but not cilostamide, in PC12 cells. Moreover, the potentiating effects of cilostazol on NGF-induced neurite outgrowth were significantly antagonized by treatment with eEF1A1 RNAi, but not the negative control of eEF1A1. These findings suggest that eEF1A1 and several common cellular signaling pathways might play a role in the mechanism of cilostazol-induced neurite outgrowth. Therefore, agents that can increase the eEF1A1 protein may have therapeutic relevance in diverse conditions with altered neurite outgrowth.

## Introduction

Cilostazol, a potent inhibitor of phosphodiesterase type-3 (PDE3), is an antiplatelet/ antithrombotic agent used worldwide for the treatment of chronic arterial occlusion and intermittent claudication with peripheral occlusion and used in Japan and some other Asian countries for the prevention of ischemic stroke [1]–[4]. The Cilostazol Stroke Prevention Study demonstrated that cilostazol significantly reduced the incidence of secondary stroke in patients with recent stroke or transient ischemic attack [5], [6]. Furthermore, subgroup analysis of this study showed that cilostazol is also useful in preventing the recurrence of vascular events in patients with lacunar infarction, and is probably effective in high-risk patients with diabetes and/or hypertension [7]. A meta-analysis of placebo-controlled randomized trials of cilostazol in patients with atherothrombosis demonstrated a significant risk reduction for cerebrovascular events, with no associated increase of bleeding risk [8]. Moreover, a randomized, double-blind study of cilostazol and aspirin demonstrated that cilostazol might be more effective and safe than aspirin for Chinese patients with ischemic stroke [9], [10]. The multicenter double-blind placebo-controlled trial showed that cilostazol prevents the progression of symptomatic intracranial arterial stenosis [11]. Very recently, the second Cilostazol Stroke Prevention Study demonstrated that cilostazol might be superior to aspirin for prevention of stroke after an ischemic stroke [12]. Taken together, these findings suggest that inhibition of PDE3 by cilostazol may contribute to its beneficial effects in these diseases although the precise mechanisms underlying the beneficial effects of cilostazol are not fully understood.

Recently, we reported that cilostazol was effective for both N-methyl-D-aspartate (NMDA) receptor antagonist phencyclidine-induced cognitive deficits and NMDA receptor antagonist dizocilpine-induced prepulse inhibition deficits in mice, suggesting that cilostazol has potential antipsychotic activity [13], [14]. There are also case reports showing that augmentation therapy with cilostazol improved the depressive symptoms in patients with geriatric depression [15], [16] and cognitive impairments in patients with moderate Alzheimer disease [17]. These findings suggest that cilostazol might have beneficial activity in the treatment of neuropsychiatric diseases. By contrast, it has been reported that mRNA levels of PDE3A and PDE3B were relatively low in the human brain whereas mRNA levels of PDE3A were the highest in the heart [18]. Thus, it is unlikely that PDE3 inhibition by cilostazol would be a major contributing factor to its effects on the brain.

The purpose of this study was to examine the precise mechanisms underlying the beneficial effects of cilostazol. First, we examined the effects of cilostazol and the other PDE3 inhibitors cilostamide and milrinone [19] on nerve growth factor (NGF)-induced neurite outgrowth in PC12 cells, which has been widely used as a model for studying neurite outgrowth [20]–[23]. In this study, we found that cilostazol, but not cilostamide or milrinone, significantly potentiated NGF-induced neurite outgrowth. Second, we examined the precise cellular mechanisms underlying the potentiation by cilostazol of NGF-induced neurite outgrowth. Finally, we identified that eukaryotic translation elongation factor eEF1A1, one of the most abundant protein synthesis factors [24], might be a novel target for cilostazol.

## Results

### Effects of three PDE3 inhibitors on NGF-induced neurite outgrowth in PC12 cells

**Figure 1:**
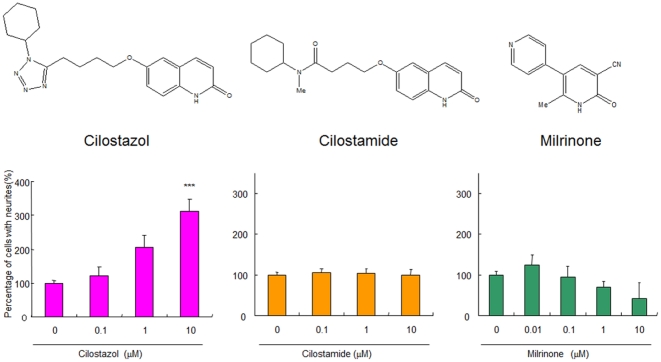
Effects of cilostazol, cilostamide, and milrinone on NGF-induced neurite outgrowth in PC12 cells. Cilostazol, but not cilostamide and milrinone, significantly increased the number of cells with neurite, in a concentration-dependent manner. Number is the concentration (µM) of drugs. ***P<0.001 as compared with control (NGF (2.5 ng/ml) alone group). The data show the mean ± SEM (n  =  6–16).

Cilostazol (0.1, 1.0 or 10 µM) significantly increased the number of cells with neurites induced by NGF (2.5 ng/ml), in a concentration-dependent manner (Fig. 1). In contrast, cilostamide (0.1, 1.0 or 10 µM) and milrinone (0.1, 1.0 or 10 µM) did not increase the number of cells with NGF (2.5 ng/ml)-induced neurites (Fig. 1). The microtubule-associated protein 2 (MAP-2) immunocytochemistry showed that cilostazol (10 µM) but not cilostamide (10 µM) increased the number of cells with NGF (2.5 ng/ml)-induced neurites (Fig. S1). These findings suggest that the inhibition of PDE3 does not contribute to the active mechanism of cilostazol.

### Role of signaling molecules proximal to TrkA in the potentiation of NGF-induced neurite outgrowth by cilostazol

**Figure 2:**
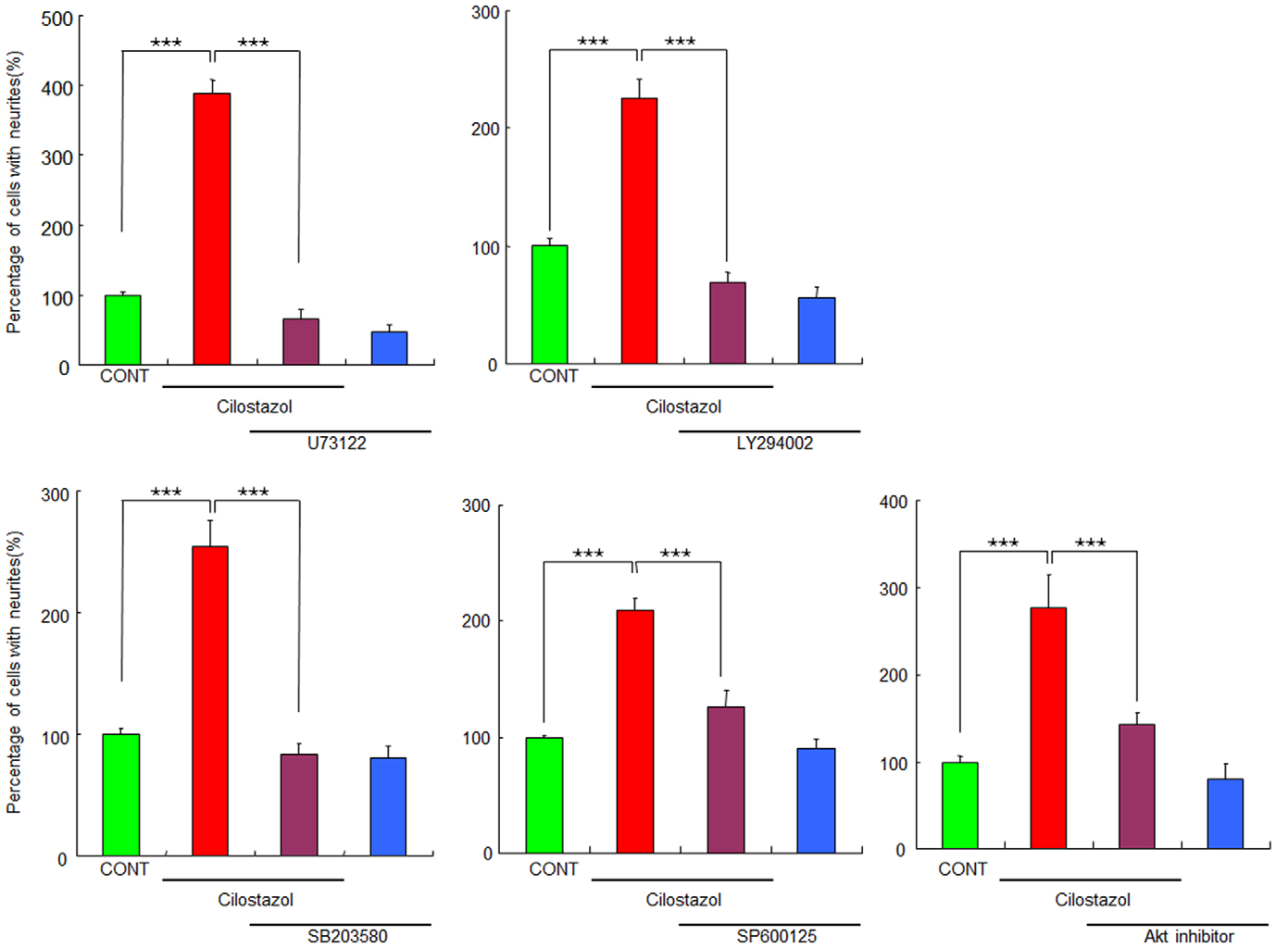
Effects of the specific inhibitors of PLC-γ, PI3K, Akt, p38MAPK, and JNK on potentiation of NGF-induced neurite outgrowth by cilostazol. The potentiating effects of cilostazol (10 µM) on the NGF (2.5 ng/ml)-induced neurite outgrowth were antagonized by co-administration of the PLC-γ inhibitor (U73122; 1.0 µM), the PI3K inhibitor (LY294002; 10 µM), the Akt inhibitor (1.0 µM), the p38MAPK inhibitor (SB203580; 10 µM), or the JNK inhibitor (SP600125; 10 µM). ***P<0.001 as compared with control (NGF (2.5 ng/ml) alone group). The data show the mean ± SEM (n  =  6–25).

We examined the effects of the specific inhibitors of PLC-γ, PI3K, Akt, p38 MAPK, and c-Jun N-terminal kinase (JNK), since these signaling molecules are activated upon the addition of NGF [20]–[23], [25]–[27]. The PLC-γ inhibitor (U73122; 1.0 µM), PI3K inhibitor (LY294002; 10 µM), Akt inhibitor (1.0 µM), p38 MAPK inhibitor (SB203580; 10 µM), and JNK inhibitor (SP600125; 10 µM) significantly blocked the potentiation of NGF-induced neurite outgrowth by cilostazol (10 µM) (Fig. 2). In contrast, these inhibitors alone did not alter NGF-induced neurite outgrowth in PC12 cells (Fig. 2).

### Role of the Ras/Raf/ERK/MAPK pathway in the potentiation of NGF-induced neurite outgrowth by cilostazol

**Figure 3:**
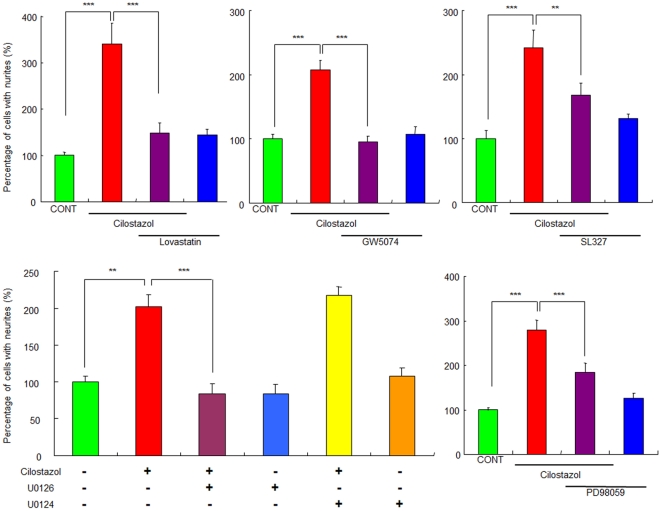
Effects of the specific inhibitors of Ras, Raf, MEK, MEK1/2, and MAPK on potentiation of NGF-induced neurite outgrowth by cilostazol. The potentiating effects of cilostazol (10 µM) on the NGF-induced neurite outgrowth were antagonized by co-administration of the Ras inhibitor (GW5074; 1.0 µM), the Raf inhibitor (lovastatin; 10 µM), the MEK inhibitor (U0126; 10 µM), the MEK1/2 inhibitor (SL327; 10 µM), and the MAPK inhibitor (PD98059; 10 µM). In contrast, U0124 (10 µM), an inactive analog of U0126, did not alter the potentiation of NGF-induced neurite outgrowth by cilostazol. **P<0.01, ***P<0.001 as compared with control (NGF (2.5 ng/ml) alone group). The data show the mean ± SEM (n  =  6–14).

The Ras/Raf/ERK/MAPK pathway is known to be involved in NGF-induced neurite outgrowth [20], [22], [23], [25], [26]. Therefore, we examined the effects of the pathway's specific inhibitors. The Ras inhibitor (GW5074; 1.0 µM), Raf inhibitor (lovastatin; 10 µM), MEK inhibitor (U0126; 10 µM), MEK1/2 inhibitor (SL327; 10 µM), and MAPK inhibitor (PD98059; 10 µM) significantly blocked the potentiation of NGF-induced neurite outgrowth by cilostazol (10 µM) (Fig. 3). In contrast, U0124 (10 µM), an inactive analog of U0126, did not alter the potentiation of NGF-induced neurite outgrowth by cilostazol (Fig. 3). Furthermore, these inhibitors alone did not alter the NGF-induced neurite outgrowth in PC12 cells (Fig. 3).

### Role of IP_3_ receptors in the potentiation of NGF-induced neurite outgrowth by cilostazol

**Figure 4:**
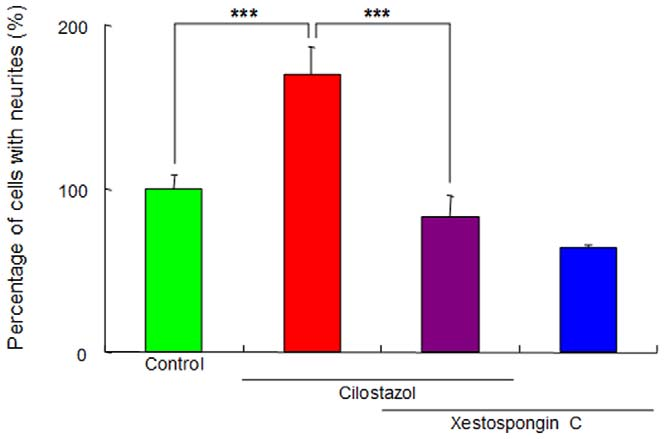
Effects of the IP_3_ receptor antagonist on potentiation of NGF-induced neurite outgrowth by cilostazol. The potentiating effects of cilostazol (10 µM) on the NGF-induced neurite outgrowth were antagonized by co-administration of the selective IP_3_ receptor antagonist xestospongin C (1.0 µM). In contrast, xestospongin C (1.0 µM) alone did not alter NGF-induced neurite outgrowth. The data show the mean ± SEM (n  =  6–12). ***P<0.001 as compared with control (NGF (2.5 ng/ml) alone group).

Previously, we reported that the receptors of the endoplasmic reticulum (ER) protein inositol 1,4,5-triphosphate (IP_3_) play a role in the NGF-induced neurite outgrowth in PC12 cells [20]–[23]. To investigate the role of IP_3_ receptors in cilostazol's action on NGF-induced neurite outgrowth, we examined the effects of xestospongin C (a selective, reversible, and membrane-permeable inhibitor of IP_3_ receptors) [28] on the effects of cilostazol on NGF-induced neurite outgrowth. Co-administration of xestospongin C (1.0 µM) significantly blocked the potentiation of NGF-induced neurite outgrowth by cilostazol (10 µM) (Fig. 4). Furthermore, administration of xestospongin C (1.0 µM) alone did not alter NGF-induced neurite outgrowth in PC12 cells (Fig. 4).

### Role of eEF1A1 in the potentiation of NGF-induced neurite outgrowth by cilostazol

To determine the molecular target of cilostazol's action on NGF-induced neurite outgrowth, we performed two-dimensional gel electrophoresis proteome analysis. We identified the eukaryotic translation elongation factor eEF1A1 as showing different protein levels in PC12 cells treated with cilostazol (10 µM) or cilostamide (10 µM); namely, eEF1A1 protein was significantly increased by the treatment with cilostazol but not by cilostamide (Fig. S2).

**Figure 5:**
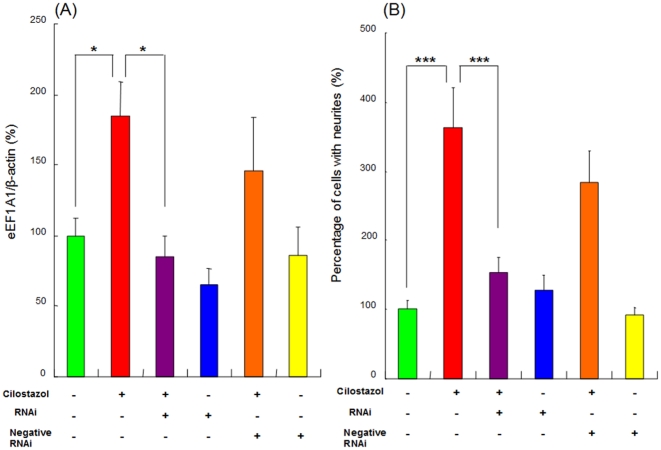
Increase in eEF1A1 protein is required for cilostazol-induced potentiation of NGF-induced neurite outgrowth in PC12 cells. (A) The potentiating effects of cilostazol (10 µM) on the eEF1A1 protein levels were significantly antagonized by treatment of eEF1A1 RNAi, but not negative RNAi. In contrast, eEF1A1 RNAi or negative RNAi alone did not alter the levels of eEF1A1 protein in the control (NGF (2.5 ng/ml)-treated) group. The data show the mean ± SEM (n  =  7–16). *p<0.05 as compared with cilostazol (10 µM) group. (B) The potentiating effects of cilostazol (10 µM) on the NGF-induced neurite outgrowth were significantly antagonized by treatment of eEF1A1 RNAi, but not negative RNAi. In contrast, eEF1A1 RNAi or negative RNAi alone did not alter NGF (2.5 ng/ml)-induced neurite outgrowth. The data show the mean ± SEM (n  =  8). ***p<0.001 as compared with cilostazol (10 µM) group.

To determine whether eEF1A1 mediates the potentiation of NGF-induced neurite outgrowth by cilostazol, we treated PC12 cells with eEF1A1 RNA interference (RNAi), which reduces the expression of the eEF1A1 protein. As shown in Fig. 5A, the increase of eEF1A1 protein by cilostazol (10 µM) was significantly blocked by treatment with eEF1A1 RNAi, but not by the negative control of eEF1A1 RNAi. In contrast, treatment with eEF1A1 RNAi or the negative control of eEF1A1 RNAi alone did not alter the basal levels of eEF1A1 protein (Fig. 5A). Furthermore, the potentiating effects of cilostazol (10 µM) on NGF-induced neurite outgrowth were significantly antagonized by treatment with eEF1A1 RNAi, but not by the negative control of eEF1A1 (Fig. 5B). In contrast, treatment with eEF1A1 RNAi or the negative control of eEF1A1 RNAi alone did not alter the NGF-induced neurite outgrowth in PC12 cells (Fig. 5B).

## Discussion

The major findings of this study are that an increase in the eEF1A1 protein by cilostazol might be involved in the mechanisms of potentiation of NGF-induced neurite outgrowth by cilostazol. First, we found that cilostazol, but not cilostamide or milrinone, could potentiate NGF-induced neurite outgrowth in PC12 cells, suggesting that inhibition of PDE3 by cilostazol might not be involved in the active mechanism for potentiation of NGF-induced neurite outgrowth by this drug. Second, the IP_3_ receptors and several common cellular signaling pathways might be involved in this action of cilostazol. Third, we identified eEF1A1 as a novel target for cilostazol. To our knowledge, this is the first report demonstrating that an increase in eEF1A1 protein by cilostazol is required for cilostazol's action on the neurite outgrowth.

NGF binds to the high-affinity tyrosine receptor TrkA, initiating several signaling pathways affecting both morphological and transcriptional targets [20], [22], [23], [25], [26]. The signaling molecules, including PLC-γ, PI3K, Akt, p38 MAPK, and JNK, are activated upon the addition of NGF [29]. PLC-γ catalyzes the hydrolysis of phosphatidylinositol-4,5-bisphosphate (PIP_2_) to diacylglycerol (DAG) and IP_3_. DAG activates protein kinase C, and IP_3_ promotes transient release of Ca^2+^ from the ER via stimulation at the IP_3_ receptors. Thus, the pathway via PLC-γ is responsible for NGF-induced neurite outgrowth [20], [22], [23], [30]. Furthermore, stimulation of PI3K is reported to be involved in the promotion of neurite outgrowth in PC12 cells [20], [22], [23], [31]. In this study, we found that the PLC-γ inhibitor U73122, the PI3K inhibitor LY294002, and an Akt inhibitor significantly blocked the potentiation of NGF-induced neurite outgrowth by cilostazol. Moreover, we found that both the p38MAPK inhibitor SB203580 and the JNK inhibitor SP600125 significantly blocked the potentiation of NGF-induced neurite outgrowth by cilostazol. Additionally, we found that the specific inhibitors for the Ras/Raf/MEK/MAPK pathways significantly blocked the potentiation of NGF-induced neurite outgrowth by cilostazol. Taken together, these findings suggest that common pathways, including PLC-γ, PI3K, Akt, p38MAPK, JNK and Ras/Raf/MEK/MAPK, are involved in the mechanisms of cilostazol's potentiation of NGF-induced neurite outgrowth. The present results may be of special interest in relation to the role of the PI3K/Akt/ERK/MAPK signaling pathway in the control of protein synthesis-dependent learning and memory [32].

It is known that IP_3_ is a ubiquitous second messenger responsible for the release of Ca^2+^ from the ER, and that control of Ca^2+^ by IP_3_ receptors on the ER is critically important in maintaining a number of cellular functions, including cell growth, neurite outgrowth [33], [34]. Interestingly, it has been reported that calcium signaling mediated by IP_3_ receptors resulted in neurite outgrowth, suggesting that IP_3_-mediated Ca^2+^ release from internal stores is necessary to maintain [Ca^2+^]_i_, within the optimum range of neurite outgrowth [35]. In this study, we found that the IP_3_ receptor antagonist xestospongin C significantly blocked the potentiation of NGF-induced neurite outgrowth by cilostazol, suggesting the role of IP_3_ receptors on NGF-induced neurite outgrowth. Previously, we reported that IP_3_ receptors play a role in the potentiation of NGF-induced neurite outgrowth by sigma-1 receptor agonists (e.g., fluvoxamine, donepezil), the ROCK inhibitor Y-27632 or the antibiotic drug minocycline [20]–[23]. Together, it seems that stimulation at the IP_3_ receptors on the ER is involved in the mechanism underlying the potentiation of NGF-induced neurite outgrowth by cilostazol.

Protein synthesis (or translation) in eukaryotic cells is fundamental for gene expression and is tightly controlled by three fundamental stages: translation, elongation, and termination [24], [36], [37]. Translation elongation requires several proteins called eukaryotic elongation factors (eEFs). Of these, eEF1A1 is one of the most abundant protein synthesis factors, and is responsible for the delivery of all aminoacyl-tRNAs to the ribosome, aside from initiator and selenocysteine tRNAs [24]. In the present study, we found that the increase in the levels of eEF1A1 protein by cilostazol might play a role in the mechanism of potentiation of NGF-induced neurite outgrowth by this drug although the precise mechanisms underlying the cilostazol-induced increase of eEF1A1 are currently unclear. It has been reported that the levels of eEF1A correlate with the rate of apoptosis upon serum withdrawal [38], and that eEF1A promotes survival following growth factor withdrawal [39], suggesting that eEF1A has neuroprotective effects. Protein synthesis is also known to be necessary for neurite outgrowth in PC12 cells [40]. Taken together, it is likely that eEF1A families including eEF1A1 play a role in neurite outgrowth, indicating that eEF1A1 may be a potential target for developing therapeutic drugs for certain neurodegenerative and psychiatric diseases. Therefore, agents that can increase the eEF1A1 protein may have therapeutic relevance in diverse conditions with altered neurite outgrowth.

Previously, we reported that an increase in the translation initiation factors eIF4AI by the antibiotic drug minocycline might play a role in the mechanisms of its action for NGF-induced neurite outgrowth in PC12 cells [23]. However, we found that cilostazol did not affect the levels of eIF4AI in PC12 cells (Fig. S3). Therefore, it is likely that an increase of eEF1A1, but not eIF4AI, by cilostazol plays a major role in the mechanism of its action.

It is known that PDE3A had a strikingly selective distribution with 10–15 fold higher levels in the human heart compared to any other tissues and tenfold higher expression than any other PDEs in the heart [18]. Furthermore, Sun et al. [41] reported that PDE3A knockout mice were protected against collagen/epinephrine-induced pulmonary thrombosis and death, and that these showed an increased heart rate, suggesting that PDE3A plays a role in regulating intracellular cAMP levels in the cardiovascular system. Considering the beneficial effects of cilostazol on neurite outgrowth, it is possible that cilostazol may have a potential therapeutic activity in heart disease.

In conclusion, the present results suggest that cilostazol, but not cilostamide and milrinone, could potentiate NGF-induced neurite outgrowth in PC12 cells, and that interaction with IP_3_ receptors and several cellular signaling pathways are involved in the mechanism underlying the pharmacological action of cilostazol. Furthermore, we identified eEF1A1 as a novel target for mechanisms of action of cilostazol. These findings offer new approaches to develop potential therapeutic drugs that can target translation elongation factors including eEF1A1.

## Materials and Methods

### Drugs

The drugs were obtained from the following sources: cilostazol (Otsuka Pharmaceutical Co., Ltd, Tokyo, Japan); cilostamide, milrinone, xestospongin C (Wako Pure Chemicals Inc., Tokyo, Japan); LY294002 (Sigma-Aldrich, St Louis, MO); NGF (Promega, Madison, WI); lovastatin, PD98059, GW5074, SB203580, MEK 1/2 inhibitor (SL327), SP600125, U0126, U0124 (Calbiochem-Novabiochem, San Diego, CA), and Akt inhibitor (Bio Vision Inc., CA). Other drugs were purchased from commercial sources.

### Cell culture

PC12 sells (RIKEN Cell Bank, Tsukuba, Japan) were cultured at 37°C, 5% CO_2_ with Dulbecco's modified Eagle's medium (DMEM) supplemented with 5% heat-inactivated fetal bovine serum (FBS), 10% heat-inactivated horse serum, and 1% penicillin. The medium was changed two or three times a week. PC12 cells were plated onto 24-well tissue culture plates coated with poly-D-lysine/laminin. Cells were plated at relatively low density (0.25×10^4^ cells/cm^2^) in DMEM medium containing 0.5% FBS, 1% penicillin streptomycin. Medium containing a minimal level of serum (0.5% FBS) was used as previously reported [22]–[23]. Previously, we examined the optimal concentration of NGF for NGF-induced neurite outgrowth in PC12 cells. NGF (2.5, 5, 10, 20, 40 ng/ml) increased the number of cells with neurite outgrowth in PC12 cells, in a concentration-dependent manner [20]. In the present studies, 2.5 ng/ml of NGF was used to study the potentiating effects of PDE3 inhibitors on NGF-induced neurite outgrowth. Twenty-four hours after plating, the medium was replaced with DMEM medium containing 0.5% FBS and 1% penicillin streptomycin with NGF (2.5 ng/ml) with or without several drugs.

### Quantification of neurite outgrowth

Four days after incubation with NGF (2.5 ng/ml) with or without the several drugs, morphometric analysis was performed on digitized images of live cells taken under phase-contrast illumination with an inverted microscope linked to a camera. Images of three fields per well were taken, with an average of 100 cells per field. Differentiated cells were counted by visual examination of the field; only cells that had at least one neurite with a length equal to the cell body diameter were counted, and were then expressed as a percentage of the total cells in the field. The counting was performed in a blinded manner.

### Differential in two-dimensional gel electrophoresis and MALDI-TOF MS analysis

In the presence of NGF (2.5 ng/ml), PC12 cells were treated with cilostazol (10 µM), or cilostamide (10 µM). After four days, cells were suspended in Laemmli lysis buffer, and two-dimensional gel electrophoresis was performed. The spots of interest were analyzed using MALDI-TOF MS (Voyager-DE STR, Applied Biosystem, CA).

### Western blot analysis

PC12 cells were washed with PBS and lysed in Laemmli lysis buffer. Aliquots (30 µg) of the proteins were measured by DC protein assay kit (Bio-Rad, Hercules, CA) and incubated for 5 min at 95°C with an equal volume of 125 mM Tris/HCl, pH 6.8, 20% glycerol, 0.1% bromphenol blue, 10% β-mercaptoethanol, 4% SDS, and subjected to SDS-PAGE using 7.5% mini-gels (Mini ProteanII; Bio-Rad, Hercules, CA). Proteins were transferred onto PVDF membranes using a Trans Blot Mini Cell (Bio-Rad, Hercules, CA). For immunodetection, the blots were blocked for 1 h in TBST (50 mM Tris/HCl, pH 7.8, 0.13 M NaCl, 0.1% Tween 20) containing 5% nonfat dry milk at room temperature (RT), followed by incubation with rabbit anti-eEF1A1 antibody (1∶250, ab37969, Abcam, Cambridge, UK) overnight at 4°C in TBST/5% blocker. The blots were washed five times with TBST. Incubation with the secondary antibody (GE Healthcare Bioscience, UK) was performed for 1 h at RT. After extensive washing, immunoreactivity was detected by ECL plus Western Blotting Detection system (GE Healthcare Bioscience, UK). Images were captured using a Fuji LAS3000-mini imaging system (Fujifilm, Tokyo, Japan) with the Multi Gauge software (Ver.3.0; Fujifilm, Tokyo, Japan) and immunoreactive bands were quantified. β-actin immunoreactivity was used to monitor equal sample loading.

### RNAi transfection

RNAi gene expression knockdown studies were performed using the TriFECTa RNAi kit (Integrated DNA Technologies, Coralville, CA) and corresponding protocol. Each 27 mer RNAi duplex was transfected into cells using Lipofectamine 2000 reagent (Invitrogen, Carlsbad, CA) following the manufacturer's guidelines. RNAi was purchased from Integrated DNA Technologies (Coralville, CA). The following sequences: Rattus norvegicus eukaryotic translation elongation factor 1A1 (Eef1a1), mRNA GenBank Accession No. NM_175838 (RNC.RNAI.N175838.10.1; IDT): sense, 5′-AGGCUUCAACGUAAAGAACGUGUCT-3′; antisense, 5′-AGACACGUUCUUUACGUUGAAGCCUAC-3′ (RNC.RNAI.N175838.10.2; IDT): sense, 5′-CGAGCUUAAAGAGAAGAUCGAUCGT-3′; antisense, 5′-ACGAUCGAUCUUCUCUUUAAGCUCGGC-3′ (RNC.RNAI.N175838.10.3; IDT): sense, 5′-CCACCAUACAGUCAGAAGAGAUACG-3′; antisense, 5′-CGUAUCUCUUCUGACUGUAUGGUGGCU-3′.

### Statistical analysis

Data are expressed as means ± standard error of the mean (SEM). Statistical analysis was performed by using one-way analysis of variance (ANOVA) and the *post hoc* Bonferroni/Dunn test. *P* values less than 0.05 were considered statistically significant.

## Supporting Information

Figure S1 **Effects of cilostazol and cilostamide on MAP-2 immunocytochemistry in PC12 cells.** Cells were fixed for 30 min at room temperature with 4% paraformaldehyde then permeabilized with 0.2% Triton and blocked with 1.5% normal goat serum, 0.1% bovine serum albumin (BSA) in 0.1 M phosphate-buffer saline for 1 h to reduce nonspecific binding. Cells were incubated overnight at 4°C with anti-microtubule-associated protein 2 (MAP-2) antibodies (1∶1000 dilution in blocking solution, Chemicon International, Temecula, CA, USA). The immunolabeling was visualized with secondary antibodies conjugated to Alexa-488 (1∶1000; Invitrogen, Carlsbad, CA, USA). MAP-2 immuncytochemistry was visualized with a fluorescence microscope (Axiovert 200, Carl Zeiss, Oberkocken, Germany). Representative photographs of MAP-2 immunocytochemistry in PC12 cells. (A) Control (NGF (2.5 ng/ml) alone) (B) NGF+cilostazol (10 µM), (C) NGF+cilostamide (10 µM).
(EPS)

Figure S2 **Effects of cilostazol and cilostamide on eEF1A1 protein in PC12 cells.** PC12 cells were treated with control (NGF (2.5 ng/ml)), NGF (2.5 ng/ml)+cilostazol (10 µM) or NGF (2.5 ng/ml)+cilostamide (10 µM) for four days. Then cells were washed with PBS, and lysed in Laemmli lysis buffer. Western blot analysis was performed using rabbit anti-eEF1A1 antibody (1∶250, ab37969, Abcam, Cambridge, UK). Levels of eEF1A1 protein in PC12 cells were significantly increased by cilostazol (10 µM), but not cilostamide (10 µM). The data show the mean ± SEM (n  =  24). **P<0.05, ***p<0.001 as compared with cilostazol treated group.
(EPS)

Figure S3 **Lack of cilostazol on eIF4AI protein in PC12 cells.** PC12 cells were treated with control (NGF (2.5 ng/ml)) or NGF (2.5 ng/ml)+cilostazol (10 µM) for four days. Then cells were washed with PBS, and lysed in Laemmli lysis buffer. Western blot analysis was performed using rabbit anti-eIF4AI antibody (1∶250, ab31217, Abcam, Cambridge, UK) as reported previously [23]. Levels of eIF4AI protein in PC12 cells were not altered by cilostazol (10 µM). The data show the mean ± SEM (n  =  8).
(EPS)
